# Tuning the Electronic Properties of Two-Dimensional Lepidocrocite Titanium Dioxide-Based Heterojunctions

**DOI:** 10.1021/acsomega.3c06786

**Published:** 2023-11-16

**Authors:** Kati Asikainen, Matti Alatalo, Marko Huttula, Assa Aravindh Sasikala Devi

**Affiliations:** Nano and Molecular Systems Research Unit, University of Oulu, Oulu FI-90014, Finland

## Abstract

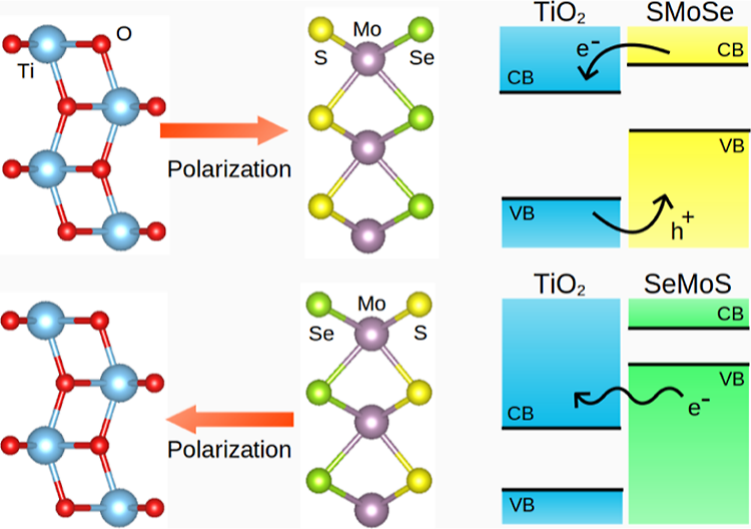

Two-dimensional (2D) heterostructures reveal novel physicochemical phenomena at different length scales that are highly desirable for technological applications. We present a comprehensive density functional theory study of van der Waals (vdW) heterostructures constructed by stacking 2D TiO_2_ and 2D MoSSe monolayers to form the TiO_2_–MoSSe heterojunction. The heterostructure formation is found to be exothermic, indicating stability. We find that by varying the atomic species at the interfaces, the electronic structure can be considerably altered due to the differences in charge transfer arising from the inherent electronegativity of the atoms. We demonstrate that the heterostructures possess a type II or type III band alignment, depending on the atomic termination of MoSSe at the interface. The observed charge transfer occurs from MoSSe to TiO_2_. Our results suggest that the Janus interface enables the tuning of electronic properties, providing an understanding of the possible applications of the TiO_2_–MoSSe heterostructure.

## Introduction

Titanium dioxide (TiO_2_) is one of the premier materials in various applications, including, e.g., photovoltaic cells,^1,2^ photocatalysis,^3,4^ and batteries.^5,6^ Excellent chemical stability, eco-friendliness, and low cost are the favorable factors of TiO_2_, but possible drawbacks that limit the performance include the large band gap of bulk phases and the fast recombination of electron and hole pairs. Various strategies, such as doping, tuning the morphology, and constructing heterostructures with different lattice-matching semiconductors, have been successfully employed in order to overcome the above shortcomings. Another strategy to enhance the activity of semiconductors is to construct low-dimensional materials that provide many active surface sites compared to their bulk counterparts. Because of this advantage, two-dimensional (2D) materials are gathering wide attention compared to their 3D phases.^7^ 2D TiO_2_ with a lepidocrocite-like structure has been synthesized through exfoliation by means of soft-chemical procedures by Sasaki et al.,^8^ and later theoretically confirmed to be thermodynamically stable.^9^ Experimentally synthesized 2D Lepidocrocite-type TiO_2_ possesses a large band gap (3.8 eV) due to quantum confinement.^10^ Despite this shortcoming, it has been shown to be a suitable candidate for both hydrogen and oxygen evolution reactions (HER and OER), with the possibility of improving the photocatalytic performance via transition-metal doping.^11,12^

For application purposes, heterojunctions constructed by stacking two or more 2D monolayers are promising, as they provide ample opportunities for band bending due to the spatial variation of the Fermi level of the semiconductors constituting the heterojunction. The three most conventional band alignments are type I (straddling gap), type II (staggered gap), and type III (broken gap). Each of these is beneficial for the development of materials for different applications. Type I band alignment is useful in optical devices such as light-emitting diodes (LEDs),^13^ as it leads to charge carrier accumulation in one location and a high recombination rate under light irradiation. By providing spatial separation of electrons and holes into different locations, thus reducing the recombination rate, type II band alignment is desirable in photocatalytic applications^14^ and photovoltaic cells.^15^ Finally, the type III band alignment allows tunneling of electrons from one material to another, making it favorable for tunnel field-effect transistors (TFETs).^16^ Multiple studies have demonstrated that vdW heterojunction formed between 2D materials can improve the light harvesting in the visible light region due to the enhanced charge transfer across the interface,^17−21^ leading to superior properties and broadening the applications of 2D materials.

2D Janus materials are a novel class of materials, extensively studied recently due to the multitude of opportunities in device applications. They were experimentally synthesized for the first time in 2017 by breaking the out-of-plane structural symmetry of MoS_2_ and replacing S atoms by Se atoms on one side.^22^ The name Janus originates from the two-faced Roman god Janus, and the MoSSe Janus material consists of two different chalcogen atoms (S and Se) on either side of a Mo atom sandwiched in the middle. Thermodynamic stability of MoSSe is well established from phonon band structure analysis,^23^ and therefore, it is worthwhile to investigate if MoSSe can form heterojunctions with other lattice-matching semiconductors. Even though the synthesis of MoSSe kick-started the research interest in these materials, recent studies have also focused on other possible materials, such as PtSSe, WSeTe, and many others,^24−27^ for various applications.

Previously, 2D lepidocrocite-type TiO_2_-based vdW heterostructures containing GaSe and MoS_2_ have been investigated for enhancing the performance of the isolated TiO_2_ monolayer.^28,29^ However, to the best of our knowledge, a vdW heterostructure of 2D TiO_2_ and 2D MoSSe has not yet been studied. These materials have fairly similar lattice parameters (*a* = 3.00 Å and *b* = 3.80 Å for 2D TiO_2_,^30^ and *a* = *b* = 3.24 Å for 2D MoSSe^22^), which is essential in creating small lattice-mismatch heterostructures. Strict lattice-matching may not be necessary,^31^ but a large mismatch can affect the stability and performance of the heterostructures. Therefore, in this work, we investigate the structural and electronic properties of the 2D TiO_2_/MoSSe vdW heterostructure by employing first-principles calculations. The study was started by optimizing the heterostructures. The electronic structure was examined through the band structure and density of states, and further, charge density analysis, planar-averaged electrostatic potential, and work function were calculated to obtain more insight into charge transport properties in the heterostructures.

## Computational Methodology

We performed first-principles density functional theory (DFT) calculations to investigate the vdW heterostructure of TiO_2_ and MoSSe using the plane wave code VASP (Vienna Ab initio Simulation Package).^32−35^ Projected augmented wave (PAW)-based pseudopotentials with plane wave basis sets were employed.^36^ A kinetic energy cutoff of 520 eV was employed to include plane waves in the basis set. The exchange–correlation potential was described by the generalized gradient approximation (GGA) in the Perdew–Burke–Ernzerhof (PBE) scheme.^37^ Since GGA is inadequate to describe the on-site Coulomb interaction between localized d and f electrons, we applied DFT + *U* to treat the localized Ti 3d electrons in order to obtain more realistic electronic properties. We added a correction of *U*_eff_ = 4.5 eV (*U* = 4.5, *J* = 0)^38^ according to the scheme of Dudarev et al.^39^ Van der Waals interactions between TiO_2_ and MoSSe were included with the DFT-D2 method of Tkatchenko and Scheffler.^40^ The Brillouin zone was sampled according to the Monkhost-Pack scheme^41^ and Gaussian smearing with a width of 0.05 eV was used. The convergence thresholds for energy and forces were set to 10^–6^ eV and 0.001 eV Å^–1^, respectively. We used VESTA^42^ for visualization and VASPKIT^43^ for postprocessing the outputs of the DFT calculations.

The initial structure of 2D TiO_2_ was constructed according to the structural parameters reported in ref (9). The 2D MoSSe unit cell was created from the hexagonal unit cell of MoS_2_ by replacing one S interface by Se. To sample the first Brillouin zone, *k*-point meshes of 6 × 6 × 1 and 5 × 5 × 1 were used for TiO_2_ and MoSSe monolayers, respectively. The vdW heterostructures were constructed via stacking pristine TiO_2_ and MoSSe monolayers with a rectangular supercell of sizes 1 × 3 × 1 and 1 × 2 × 1, respectively, along the vertical direction. The size of the rectangular unit cell of MoSSe was *a* = 3.25 Å and *b* = 5.64 Å (Figure S1). Due to the lattice mismatch, the constructed heterostructure forms a Moiré pattern.^44^ Resulting from this, the stacking configuration is not the same in all regions, but in a long range, the periodicity appears. Li et al. have investigated a few stacking configurations of 2D lepidocrocite-type TiO_2_ and 2D MoS_2_. They found that the Moiré pattern, in which the zigzag direction of MoS_2_ and the in-plane edge of TiO_2_ with a smaller lattice parameter were aligned in the same direction, was the most stable according to adsorption energies calculated with respect to the interlayer distance.^29^ Therefore, in this work, we focused on this particular stacking configuration of 2D TiO_2_ and 2D MoSSe (Figure S2). The lattice mismatch in the *x*- and *y*-directions was calculated as , where *a* and *b* denote the lattice parameters of TiO_2_ and MoSSe monolayers, respectively. This resulted in a lattice mismatch of 7.17% in the *x*-direction and −0.08% in the *y*-direction. In the *y*-direction, the effect of strain is negligible. A vacuum with a thickness of around 23 Å was added along the *z*-direction to both interfaces to avoid correlation between periodic images. Because the Mo layer is sandwiched by two distinct chalcogen layers, the S layer and the Se layer, heterostructures with two different interfaces can be constructed: TiO_2_–MoSSe (S atoms at the interface) and TiO_2_–MoSeS (Se atoms at the interface). A *k*-point sampling of 18 × 5 × 1 within the Monkhorst–Pack scheme was adopted.

## Results and Discussion

Before building the heterostructures, we investigated freestanding 2D TiO_2_ and MoSSe monolayers. The optimized geometries of the monolayers are shown in Figure S3. The optimized lattice parameters of TiO_2_ were *a* = 3.03 Å and *b* = 3.77 Å, and Ti–O distances were 1.85–2.22 Å (Figure S4). For MoSSe, we found lattice parameters of *a* = *b* = 3.25 Å. Mo–S, Mo–Se, Se–Se, and S–S distances were 2.42 2.54, 3.26, and 3.26 Å, respectively. Our calculated lattice parameters are in agreement with existing research.^28,45^ Furthermore, we calculated the electronic band structure of the monolayers (Figure S5). Using the GGA functional, we found a direct band gap of 2.76 eV at Γ for TiO_2_.^9,46^ Applying the Hubbard correction, a band gap of 3.30 eV was obtained, which compares better with the experimental value of 3.8 eV^10^ and the previously obtained value using the GGA + *U*.^28^ Previously, higher-level approximations were also applied to calculate the electronic structure. Using the HSE06 functional, Li et al.^29^ obtained a band gap of 3.87 eV, which is extremely close to the experimental value. Besides, Wang et al.^47^ have obtained a band gap of 5.97 eV using the GW approximation, and Zhou et al.^46^ have calculated the band gap using the G_0_W_0_+BSE and reported a band gap of 5.3 eV. Therefore, it can be seen that we need to be careful while comparing different approaches, as over- and under-estimation of band gaps can be seen across different functionals. The calculated direct band gap of 1.59 eV for MoSSe is closer to the experimental band gap of 1.68 eV^22^ and reported results using the GGA functional.^45,48^

**Figure 1:**
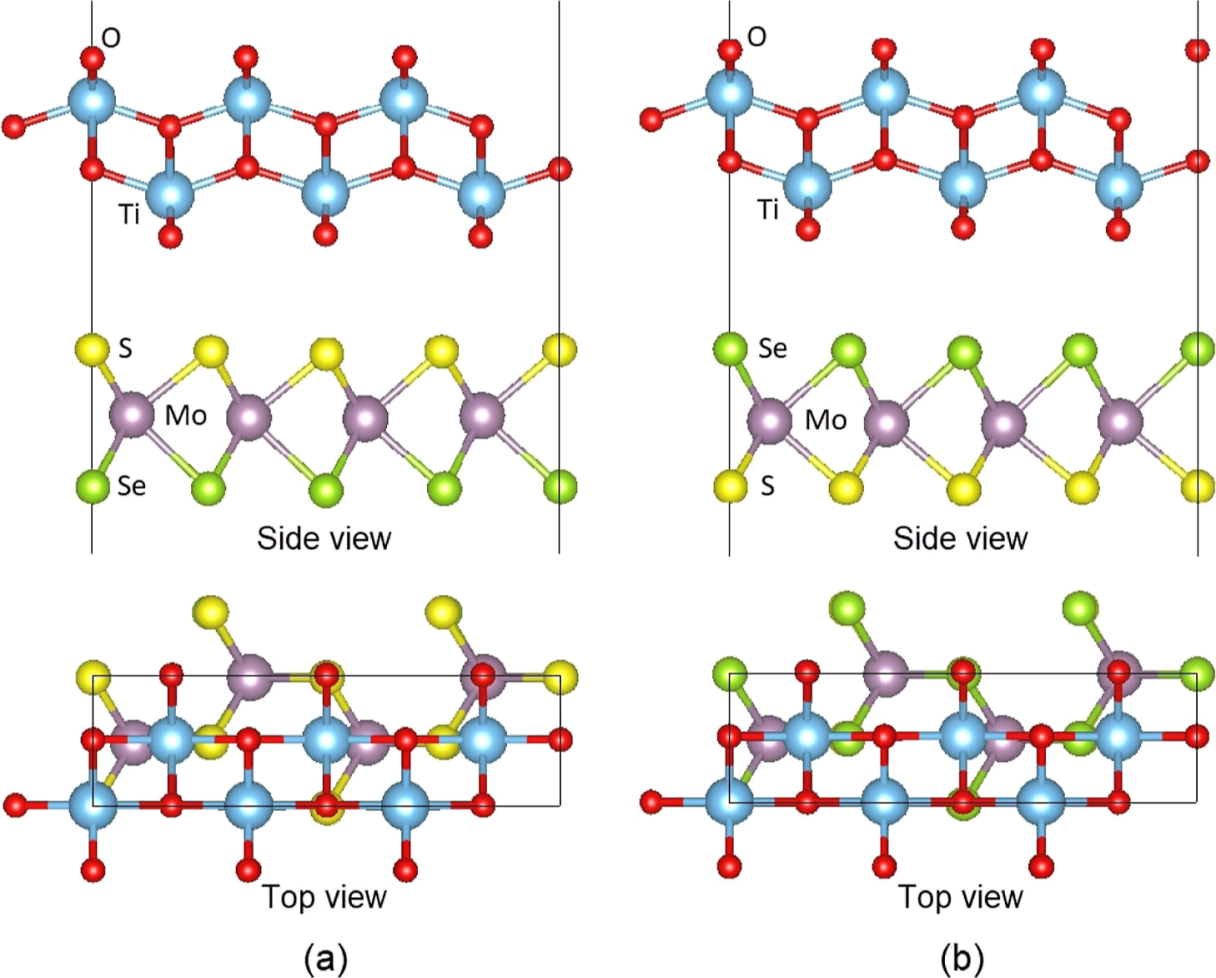
Side view (along *y*-direction) and top view (along *z*-direction) of the optimized (a) TiO_2_/MoSSe and (b) TiO_2_/MoSeS heterostructures. The color coding of the atoms is the same here and elsewhere.

The optimized structures of the TiO_2_/MoSSe and TiO_2_/MoSeS heterostructures are shown in Figure 1. The obtained lattice constants of the above two heterostructures were *a* = 3.11 and *b* = 11.18 Å after the optimization. The interlayer distance between the monolayers was 2.75 Å in the TiO_2_/MoSSe and 2.90 Å in the TiO_2_/MoSeS. These values fall within the optimal range of vdW interaction, as discussed by Wang et al.^49^ and Pushkarev et al.^50^ The smaller interlayer distance in the TiO_2_/MoSSe may be attributed to a larger covalent radius of the Se atoms than the S atoms, resulting in a larger spacing between the monolayers.^51,52^ Interlayer distances smaller than 3 Å have also been reported in previous investigations on TiO_2_-based heterostructures^28,29,53^ and other vdW heterostructures as well.^54−56^ To estimate the stability of the heterostructures, we calculated the formation energies using the equation
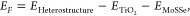
1where *E*_Heterostructure_ is the total energy of the heterostructure, and *E*_TiO_2__ and *E*_MoSSe_ are the total energies of the TiO_2_ and MoSSe monolayers, respectively. The calculated formation energies of −5.52 and −5.50 eV for TiO_2_/MoSSe and TiO_2_/MoSeS, respectively, indicated that the vdW heterostructures are energetically favorable. Previously, Ahmad et al.^57^ reported a low binding energy of −5.97 eV for the InSe/PdSe_2_ heterostructure. Together with the interlayer distances, the results suggest high stability for the heterostructure and stronger physical interaction between the monolayers.^50,51,55,56^ Of the two heterostructures, the TiO_2_/MoSSe is slightly more stable than the TiO_2_/MoSeS.

**Figure 2:**
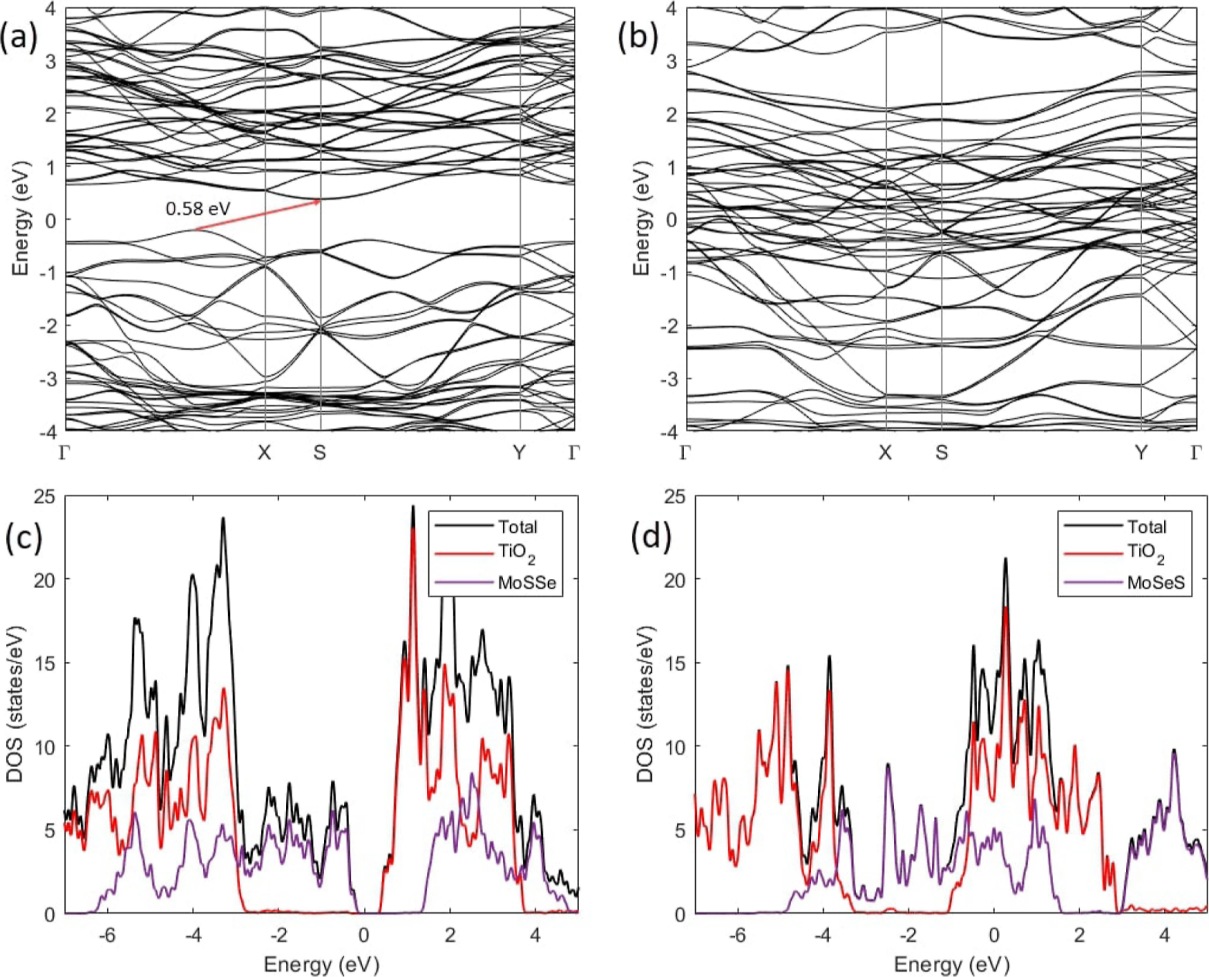
Band structure and density of states of the TiO_2_/MoSSe (a,c) and TiO_2_/MoSeS (b,d) heterostructures using the GGA + *U* functional. The indirect band gap is indicated with a red arrow in (a).

After investigating the electronic structure of the monolayers, we extended the same calculations to the heterostructures. Figure 2 shows the band structure and density of states (DOS) of the heterostructures. The formation of the vdW heterostructure led to a significant left-shift of the density of states of TiO_2_ toward the lower energy regions. The TiO_2_/MoSSe heterostructure was found to be an indirect-band gap semiconductor (Figure 2a). The valence band maximum (VBM) was located between Γ and X and the conduction band minimum (CBM) at S, resulting in a band gap of 0.58 eV. This is evidently lower than the band gap of the freestanding monolayers, facilitating electron excitation. The quasi-direct band gap was located between S and Y and was 0.84 eV. It can be seen from the DOS that the top of the valence band (VB) is dominated by the MoSSe monolayer, while the bottom of the conduction band (CB) is contributed by TiO_2_ (Figure 2c). This indicated a type II band alignment between TiO_2_ and MoSSe, allowing charge transfer from MoSSe to TiO_2_. To confirm this, we calculated the decomposed charge densities of the VBM and CBM, which showed that the VBM is composed of the states of MoSSe, while the CBM is contributed by TiO_2_ (Figure S6a,b). Consequently, the charge density is completely separated, making it possible to separate the photogenerated electrons and holes in the heterostructure. This particular band alignment is extremely desired for photocatalytic applications.^14,20^ The band structure revealed the formation of metallic states in TiO_2_/MoSeS due to the overlapping of the VB of MoSSe with the CB of TiO_2_ (Figure 2b). Both the VBM and CBM of TiO_2_ lied below the Fermi level and the VBM and CBM of MoSSe above the Fermi level (Figure 2d), that is, the highest VB edge of MoSSe was located at a higher energy level than the lowest CB edge of TiO_2_ (Figure 2d). This suggested that the TiO_2_/MoSeS heterostructure possesses a broken-gap type-III band alignment. The broken gap enables a band-to-band tunneling (BTBT) mechanism between TiO_2_ and MoSSe.^17^ Thus, electrons in the VB of MoSSe can directly tunnel to the CB of TiO_2_ without light absorption or emission. The decomposed charge density plots in Figure S6c,d showed that the VBM is contributed by MoSSe, while the CBM is distributed around both monolayers. In TiO_2_, the CBM is mainly concentrated on the atoms at the interface in MoSSe, and the charge density is distributed around all atoms in MoSSe. Electrons from the VBM of MoSSe can be excited by photons in the CBM of MoSSe. Simultaneously, the band alignment allows electron tunneling from the VBM of MoSSe to the CBM of TiO_2_, explaining the observed decomposed charge density distribution. We found a large tunneling window (energy difference between the VB of MoSSe and the CB of TiO_2_) of around 2.78 eV, indicating a greater tunneling probability for electrons. Due to the formation of type III band alignment associated with metallic character, the TiO_2_/MoSeS heterostructure can be a potential candidate for tunneling devices such as TFETs and Esaki diodes.^16,58^ The particular band alignment can induce a negative differential resistance in heterostructures, which is favorable, especially in TFETs.^59,60^ The contributions of each element to the density of states are shown in Figure S7.

**Figure 3:**
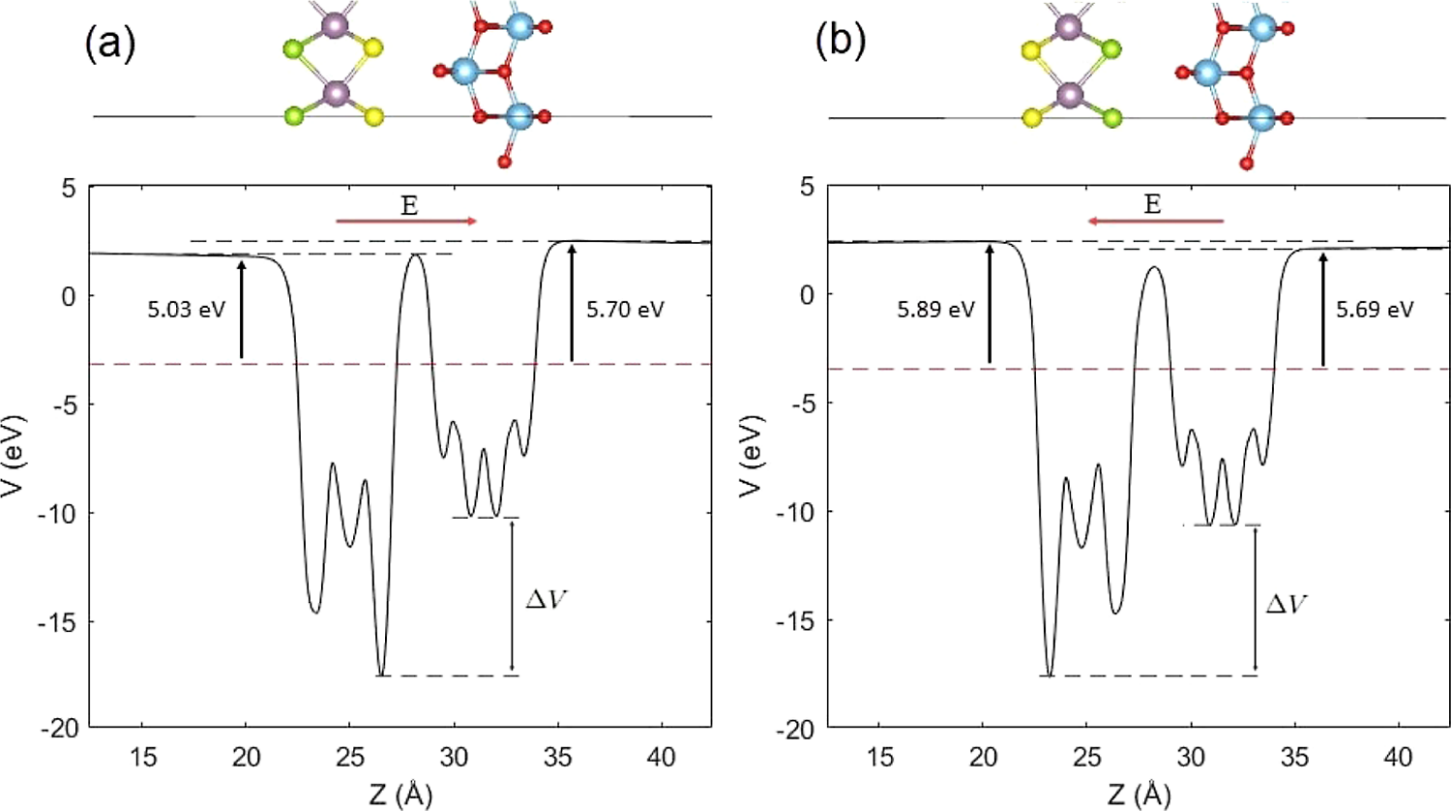
Electrostatic potential of (a) TiO_2_/MoSSe and (b) TiO_2_/MoSeS heterostructures. The Fermi level is indicated with a dashed red line, and the direction of the built-in electric field *E* is indicated with a red arrow. The potential drop of the heterostructures across the interface is represented by Δ*V*.

We calculated the work function Φ using the equation
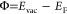
2where *E*_vac_ and *E*_F_ represent the vacuum level and Fermi level, respectively. The calculated work function of TiO_2_ was 8.60 eV (Figure S8a), whereas MoSSe exhibited two different work functions: 5.20 eV at the Se termination and 5.79 eV at the S termination due to the intrinsic polarization (Figure S8b). This resulted in an electrostatic potential difference of 0.59 eV between the terminations. These are in line with previous work.^18,61^ Because the work functions of both the S and Se terminations of MoSSe are smaller than those of TiO_2_, the results suggest electron flow from MoSSe to TiO_2_ when combining the two monolayers until thermodynamic equilibrium is reached. We found that the formation of the heterostructures significantly decreased the work function of TiO_2_. Moreover, because of the electrostatic potential difference between the terminations of MoSSe, the work functions of the two surfaces of TiO_2_/MoSSe and TiO_2_/MoSeS were found to be different. The planar-averaged electrostatic potential of the heterostructures along the *z*-direction is shown in Figure 3. The work functions at the TiO_2_ and MoSSe surfaces were 5.70 and 5.03 eV in the TiO_2_/MoSSe (Figure 3a), and 5.69 and 5.89 eV in the TiO_2_/MoSeS (Figure 3b), respectively. Interestingly, the heterostructure possesses a lower work function at the MoSSe surface when the S termination is placed at the interface, whereas placing the Se termination at the interface results in a lower work function at the TiO_2_ surface. This has also been observed in other 2D MoSSe-based heterostructures.^62−64^ This may be explained by the intrinsic polarization observed in the pristine MoSSe. Since S atoms have larger electronegativity, electrons tend to accumulate in the S layer of MoSSe, increasing the work function and potential energy (Figure S8b). Thus, the direction of the intrinsic dipole moment is from S to Se. When combining MoSSe with TiO_2_, this property appears to be preserved, showing that the intrinsic polarization of MoSSe takes up a significant role in giving rise to a polarization in the heterostructures. Resulting from the different work functions at the two surfaces, there exists an electrostatic potential difference Δϕ of 0.67 and 0.2 eV in the TiO_2_/MoSSe and TiO_2_/MoSeS, respectively, which induces a built-in electric field at the interface of the heterostructures,^18,19,65^ pointing from MoSSe to TiO_2_ in the TiO_2_/MoSSe and from TiO_2_ to MoSSe in the TiO_2_/MoSeS. Moreover, the electrostatic potential of TiO_2_ was deeper than that of MoSSe, resulting in a potential drop of Δ*V* across the interface. The potential drop was 7.37 eV in the TiO_2_/MoSSe and 6.95 eV in the TiO_2_/MoSeS. This gradient, directed from MoSSe to TiO_2_, was attributed to the difference in the electronegativity of oxygen (3.44), S (2.58), and Se (2.55), and it can further facilitate the charge separation of electrons and holes.^68,69^

**Figure 4:**
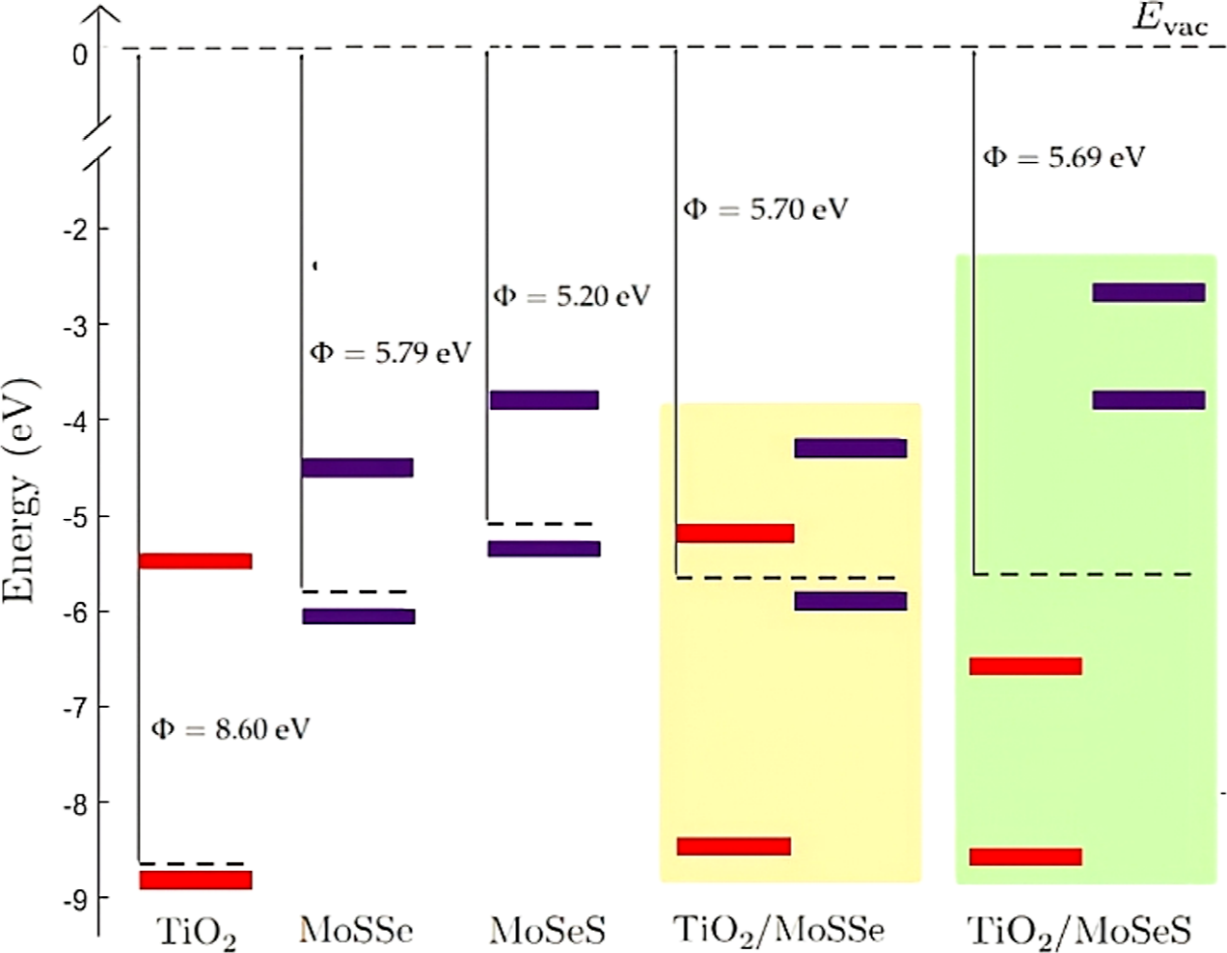
Band alignment and work function of the freestanding TiO_2_ and MoSSe monolayers and TiO_2_/MoSSe and TiO_2_/MoSeS heterostructures. The band positions of TiO_2_ are depicted in red, and those of MoSSe are in purple. The band positions of MoSSe are represented relative to the vacuum level by considering the work function of both the S-termination (MoSSe) and the Se-termination (MoSeS). The band positions of TiO_2_/MoSSe and TiO_2_/MoSeS are represented by using the work function of TiO_2_ (Figure 3). The Fermi level is indicated with a black dashed line, and the vacuum level is set to zero.

A schematic diagram in Figure 4 shows the work functions and band edge positions of the monolayers and heterostructures with respect to the vacuum level. The band alignment of materials is essential in designing materials for practical applications. The TiO_2_/MoSSe heterostructure retained the band ordering of the two monolayers, resulting in a type II band alignment, and the band gap energies of the monolayers were only slightly affected by the formation of the heterostructure. We found band gaps of 3.26 eV for TiO_2_ and 1.58 eV for MoSSe. In TiO_2_/MoSeS, the band gap of TiO_2_ and MoSSe did not overlap, which is known as a broken gap. The energy difference between the VB and CB of TiO_2_ and the VB and CB of MoSSe was reduced to 2.43 and 1.44 eV, respectively. This shows that Se termination at the interface affects more significantly the band gap energies of the monolayers in the heterostructure.

**Figure 5:**
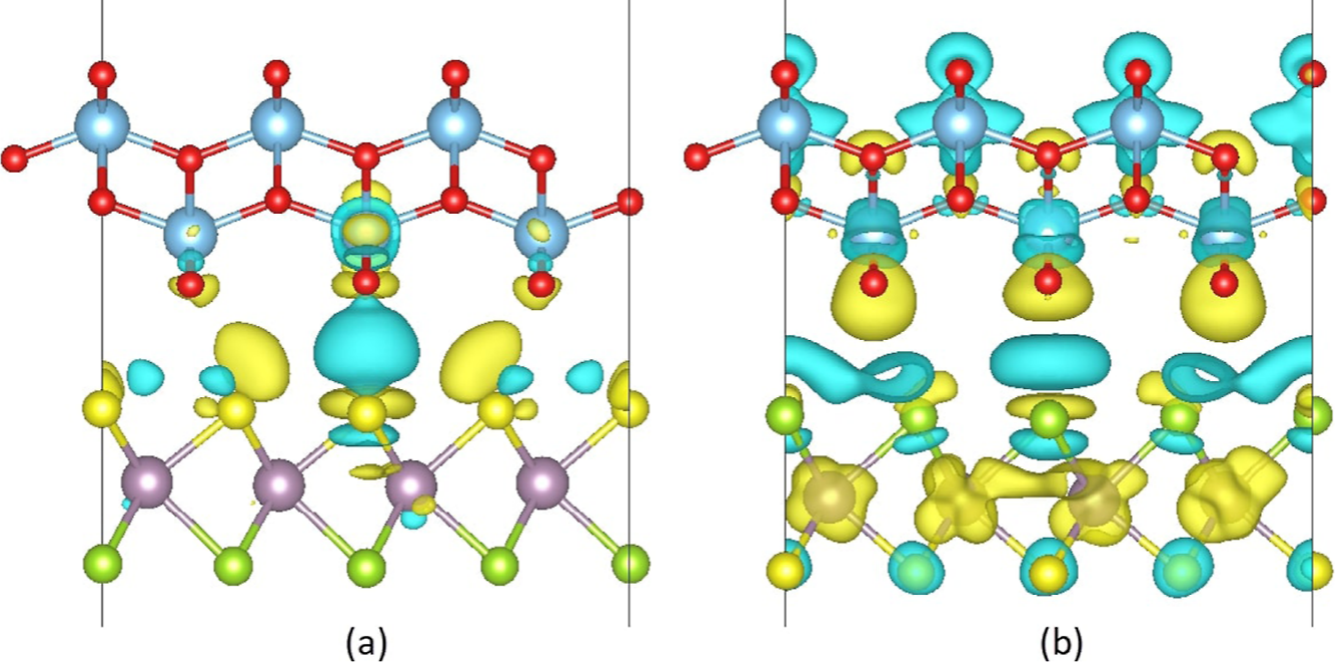
Charge density difference of (a) TiO_2_/MoSSe and (b) TiO_2_/MoSeS. The yellow isosurface refers to electron gain, and blue refers to electron loss. The isosurface value is set to 0.003 eÅ^–3^.

To further identify the charge transfer in the heterostructures, we have calculated the charge density difference in Figure 5 as Δρ = ρ_Heterostructure_ – ρ_TiO_2__ – ρ_MoSSe_, where ρ_Heterostructure_, ρ_TiO_2__, and ρ_MoSSe_ are the charge densities of the heterostructure, TiO_2_ monolayer, and MoSSe monolayer, respectively. In TiO_2_/MoSSe, the charge redistribution was localized at the interface. In TiO_2_, the O atoms at the interface mainly experienced changes in the charge density, whereas the changes were more obvious in the S termination of MoSSe. The strongest interaction occurred between the nearest O and S atoms, where the charge redistribution is significant. The results are comparable with the study conducted by Li et al., although different exchange–correlation functionals were used in the calculations.^29^ In the TiO_2_/MoSeS, notable charge rearrangement occurred around the interface, which also extended to the outer side of both monolayers. The blue isosurface at the S and Se terminations showed that MoSSe contributes electrons to TiO_2_. In order to quantify the amount of charge transfer in the heterostructures, we performed the Bader analysis.^70^ According to the analysis, a charge of 0.038 *e* and 0.020 *e* per unit cell was transferred from MoSSe to TiO_2_ in the TiO_2_/MoSSe and TiO_2_/MoSeS, respectively. Thus, after the heterostructures are constructed, n-type doping is realized in TiO_2_, while p-type doping is realized in MoSSe. The built-in electric field pointing from MoSSe to TiO_2_ facilitates the charge separation and suppresses the recombination rate of charge carriers in the TiO_2_/MoSSe.^65^ Moreover, the larger charge transfer can be attributed to the strong interlayer coupling and narrower interlayer distance, contributing to the larger amount of charge transferred from TiO_2_ to MoSSe.^66,67^ In the TiO_2_/MoSeS, electrons were transferred in the opposite direction of the built-in electric field, which is proposed to contribute to reducing charge transfer across the interface. The Bader charges of the individual atoms in the isolated monolayers and heterostructures are provided in Figures S9 and S10, confirming the charge redistribution after the heterostructures. The values support the strongest interaction between the closest O and S(Se) atoms at the interface. The small charge transfer across the interface indicates relatively little chemical interaction between TiO_2_ and MoSSe monolayers. In all, these results indicate that it is possible to construct stable heterostructures out of lattice-matching semiconductor monolayers and tune the electronic properties according to varying interface terminations ranging from semiconducting to metallic.

## Conclusions

We have investigated the structural and electronic properties of the TiO_2_/MoSSe and TiO_2_/MoSeS vdW heterostructures using first-principles calculations. Both heterostructures were energetically stable, as indicated by their negative formation energies. The band alignment was found to depend on the interface termination of MoSSe. The S termination at the interface led to a type II band alignment, providing efficient separation of photogenerated electrons and holes. The Se termination at the interface resulted in a type III band alignment and enabled the band-to-band tunneling of electrons across the interface. After forming the heterostructures, electron transfer occurred from MoSSe to TiO_2_. Interestingly, a built-in electric field was developed inside the heterostructures due to the difference in the work functions of the TiO_2_ and MoSSe layers, and that influenced the charge transfer at the interface. Our work demonstrates that the interface termination of MoSSe is a key factor in determining the properties of the TiO_2_-based vdW heterostructure. Tunability via changing the interface termination makes the heterostructure of 2D TiO_2_ and MoSSe a potential candidate for various applications.

## Supporting Information Available

Crystal structure of MoSSe and TiO_2_ monolayers, different stacking configurations of TiO_2_ and MoSSe monolayers, bond lengths in TiO_2_, band structure of MoSSe and TiO_2_, partial charge densities and partial density of states of TiO_2_/MoSSe and TiO_2_/MoSeS, planar-averaged electrostatic potential of MoSSe and TiO_2_, and Bader charges in MoSSe and TiO_2_ and in TiO_2_/MoSSe and TiO_2_/MoSSe (PDF)
