# Supplementary material for: Tuning the Electronic Properties of Two-Dimensional Lepidocrocite Titanium Dioxide-Based Heterojunctions
Source: ACS Omega. 2023 Nov 16;8(47):45056–64. doi: 10.1021/acsomega.3c06786 (PMC10688046; doi:10.1021/acsomega.3c06786)
Supplement: Supplementary file 1 — ao3c06786_si_001.pdf [file ao3c06786_si_001.pdf]

Supplementary information

Tuning the Electronic Properties of Two-Dimensional Lepidocrocite Titanium Dioxide Based Heterojunctions

Kati Asikainen†,* Matti Alatalo†, Marko Huttula†, and S. Assa Aravindh†,*

†*Nano and Molecular Systems Research Unit, University of Oulu, FI-90014, Finland*

E-mail: Kati.Asikainen@oulu.fi; Assa.SasikalaDevi@oulu.fi

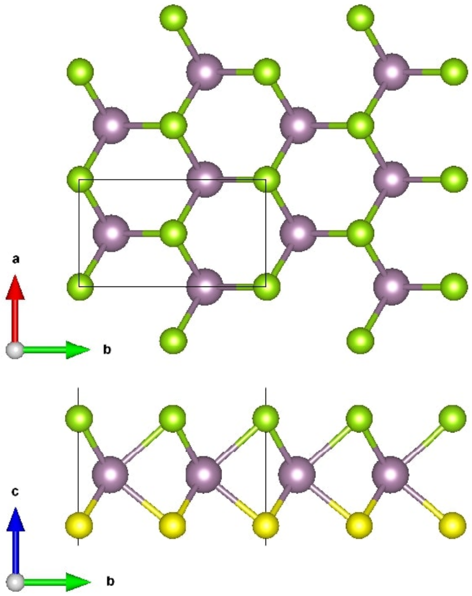

Figure S1: Rectangular unit cell of 2D MoSSe with a unit cell size of $a = 3.25$ Å and $b = 5.64$ Å.

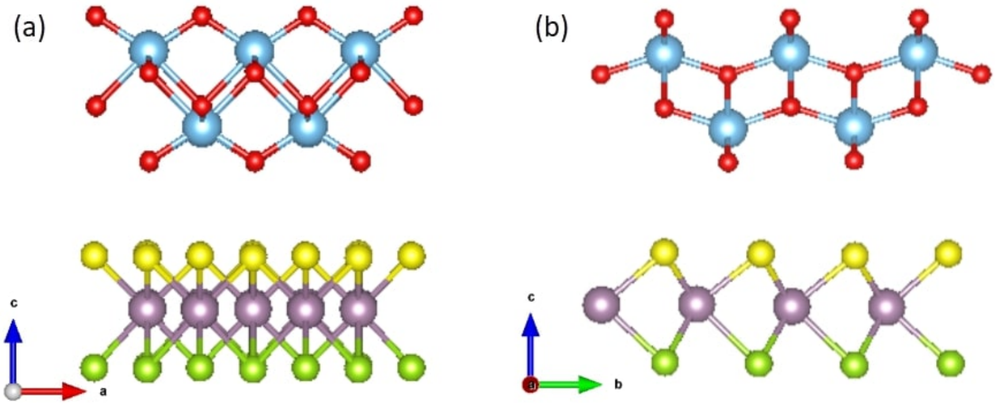

Figure S2: Stacking configuration of 2D lepidocrocite-type TiO_2 and 2D MoSSe monolayers along the a) x-direction and b) y-direction. According to Li *et al.* the particular stacking configuration of 2D TiO_2 and MoS_2 is the most stable.[1]

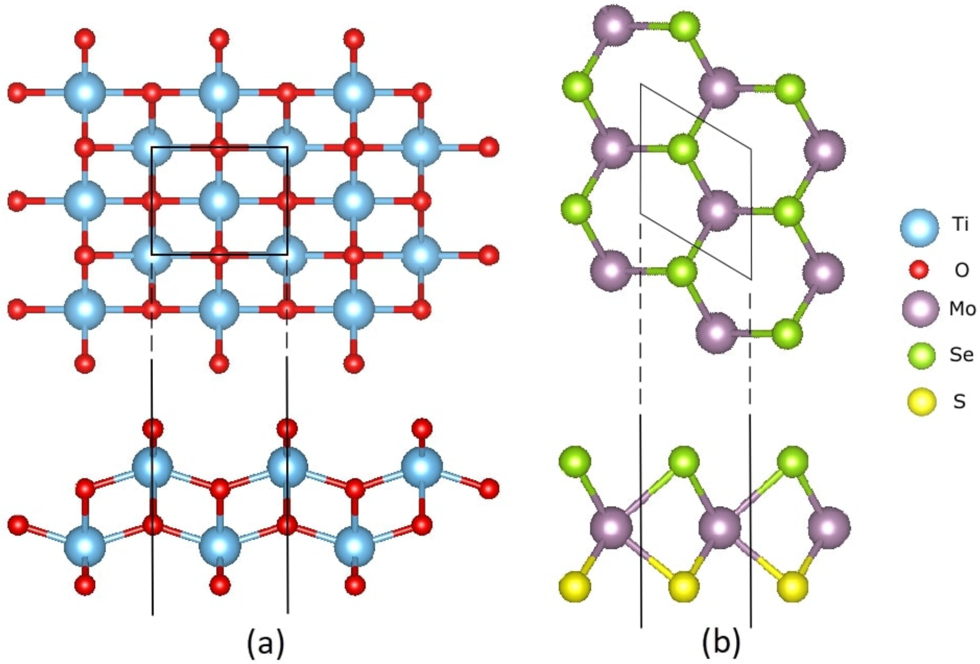

Figure S3: Top (upper) and side (lower) view of the a) 2D lepidocrocite-type TiO_2 and b) 2D MoSSe. The primitive unit cells are indicated with black lines.

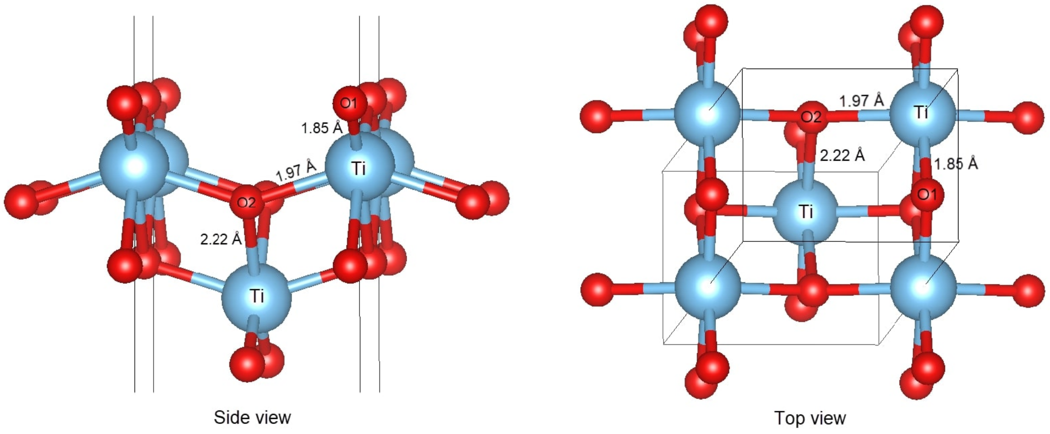

Figure S4: The optimized structure of 2D lepidocrocite-type TiO_2. The monolayer consists of two-fold (O1) and four-fold (O2) oxygen atoms, and six-fold titanium (Ti) atoms. The Ti-O1 bond lengths were found to be 1.85 Å, and the two different Ti-O2 bonds were 1.97 Å and 2.22 Å, being in agreement with reported values.[2,3]

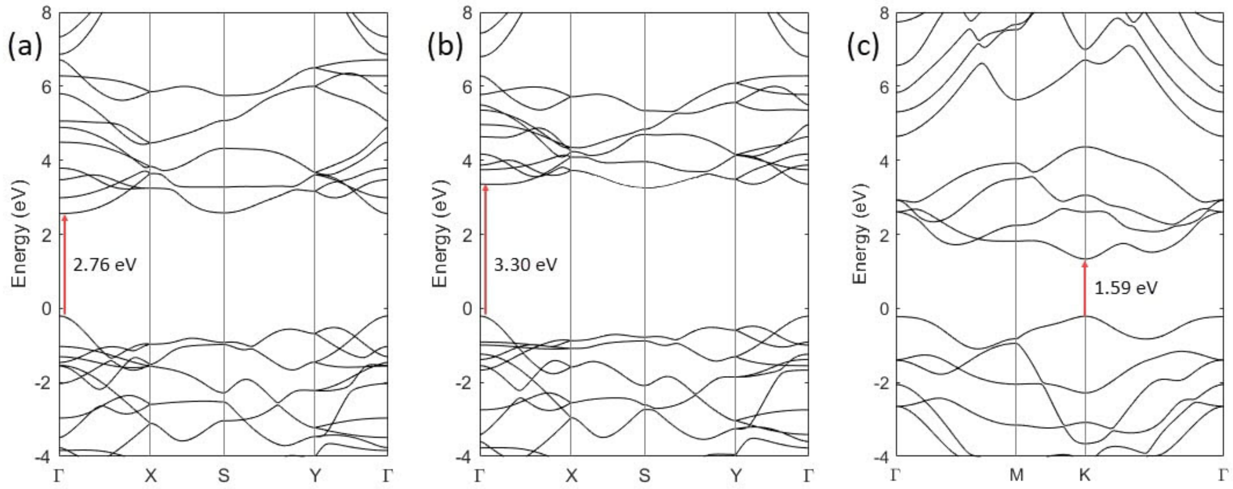

Figure S5: Band structure of 2D TiO_2 using the a) GGA and b) GGA+U functional. A direct band gap of 2.76 eV and 3.30 eV were found, respectively. For c) MoSSe we found a direct band gap of 1.59 eV using the GGA functional. Band gap is indicated with a red arrow in the plots.

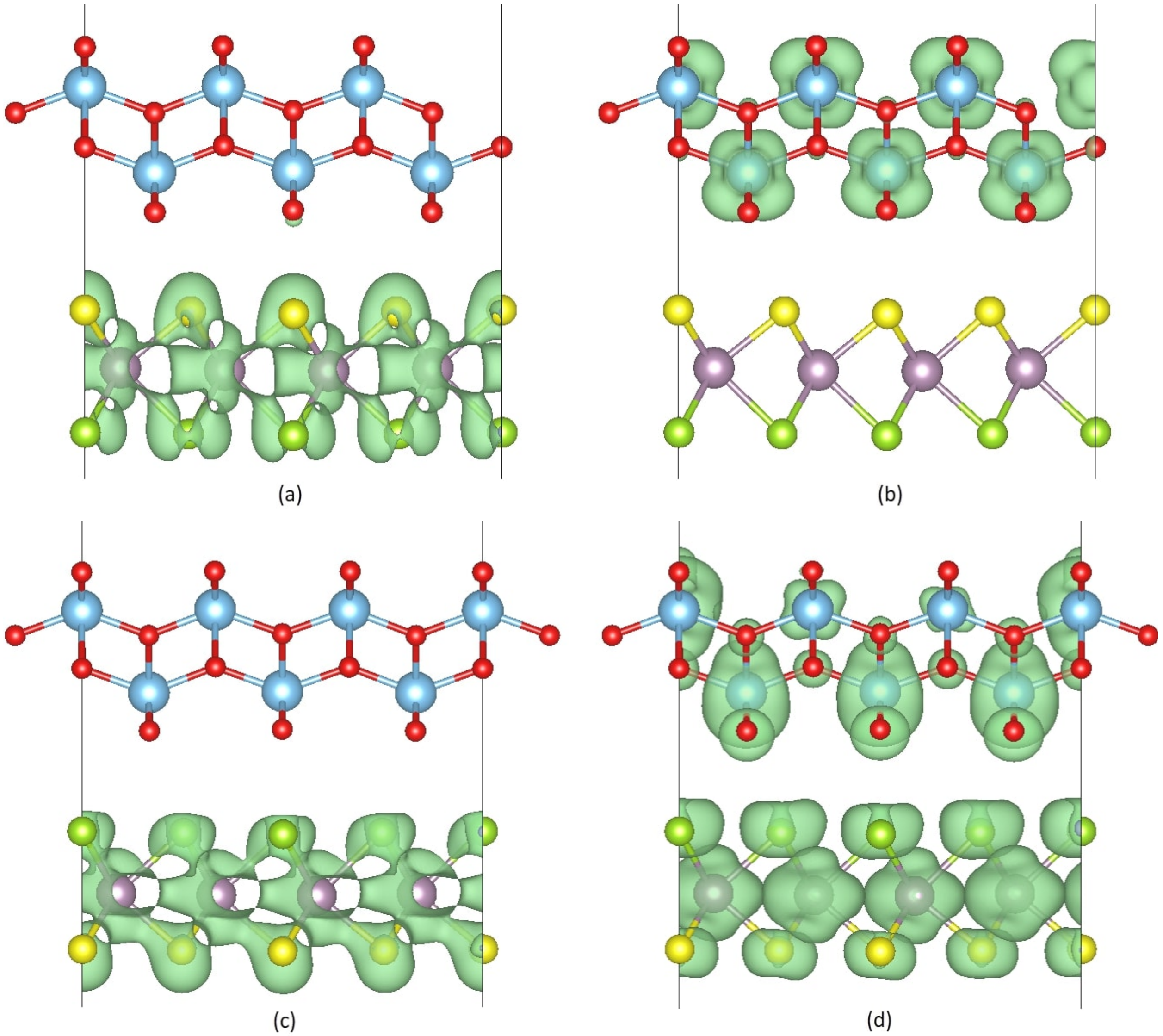

Figure S6: Decomposed charge densities of the VBM and CBM of the TiO_2/MoSSe (a and b) and TiO_2/MoSeS (c and d). Isosurface value is set to 0.006 $e\text{Å}^{-3}$.

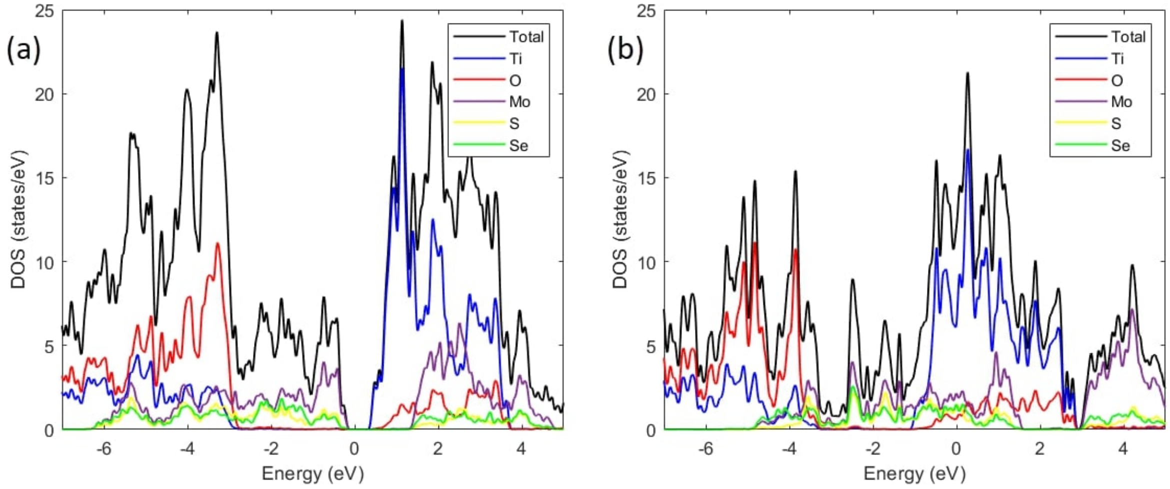

Figure S7: Partial density of states of Ti, O, Mo, S and Se in the a) TiO_2/MoSSe and b) TiO_2/MoSeS heterostructures.

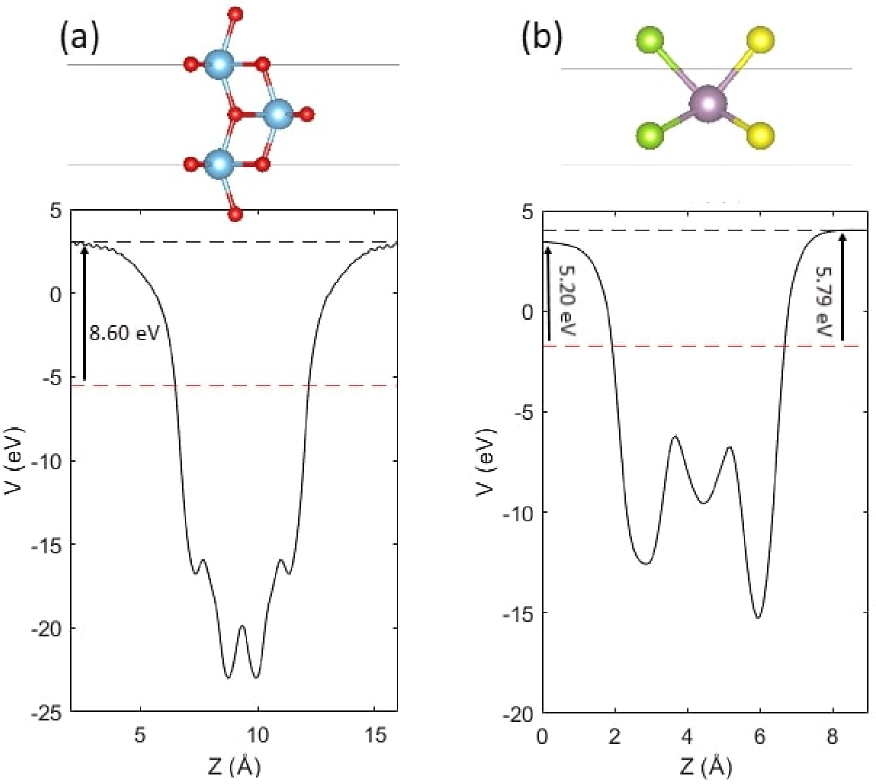

Figure S8: Planar-averaged electrostatic potential of the a) TiO_2 and b) MoSSe monolayers.

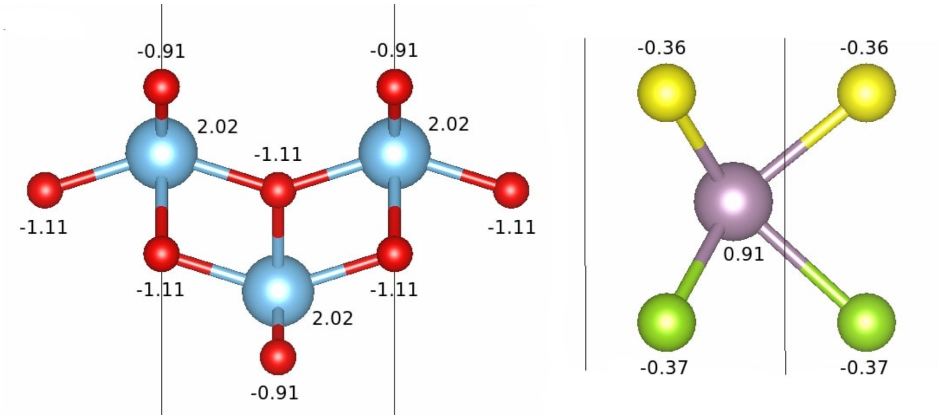

Figure S9: Bader charges of the atoms in the free-standing a) TiO_2 and b) MoSSe monolayers. Positive value refers to electron loss and negative value to electron gain. In the TiO_2 Ti atoms give 2.02 *e* for covalent bonding while two-fold and four-fold oxygen atoms gain -0.91 *e* and -1.11 *e* per unit cell, respectively. In the MoSSe Mo atom exhibits a loss of charge which is accumulated to S and Se sites.

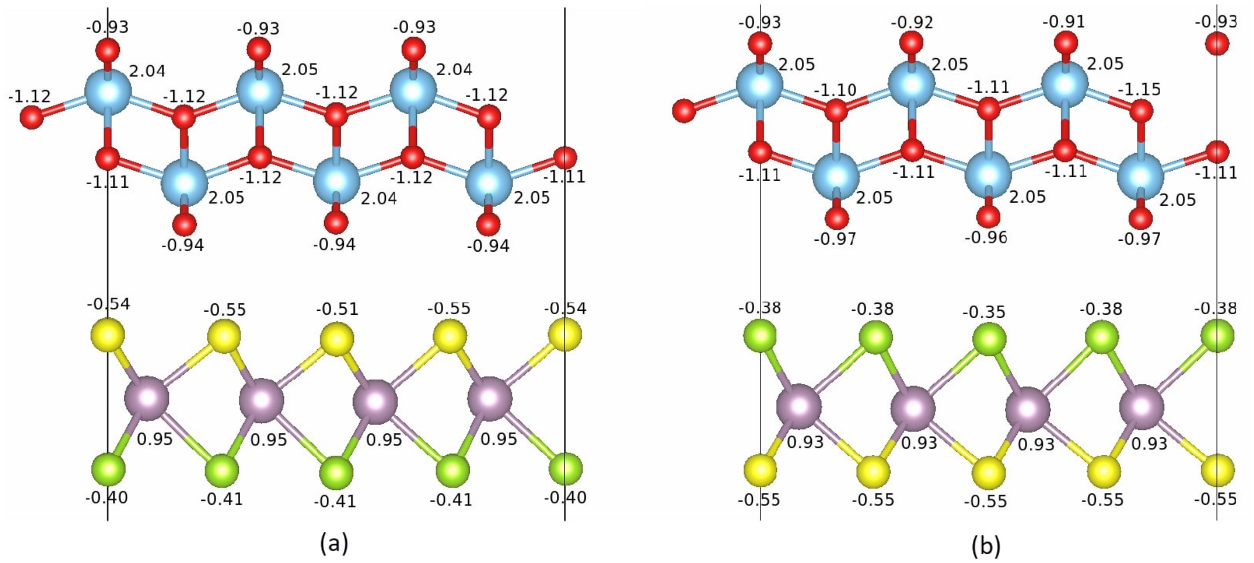

Figure S10: Bader charges of the atoms in the a) TiO_2/MoSSe and b) TiO_2/MoSeS after constructing the heterostructures. The charge redistribution occurs in both monolayers. At the interface the Bader charges of O atoms and S (Se) atoms vary, showing the strongest interaction between the closest O and S (Se) atoms.

References

(1) Li, Y.; Cai, C.; Sun, B.; Chen, J. Novel electronic properties of 2D MoS_2 /TiO_2 van der Waals heterostructure. *Semicond. Sci. Technol.* **2017**, *32*, 105011.

(2) Sato, H.; Oto, K.; Sasaki, T.; Yamagishi, A. First-Principles Study of Two-Dimensional Titanium Dioxides. *J. Phys. Chem. B* **2003**, *107*, 9824 .

(3) Zhao, Y.; Zhang, H; Cheng, X., A new type-II lepidocrocite-type TiO2/GaSe heterostructure: Electronic and optical properties, bandgap engineering, interaction with ultrafast laser pulses. *arXiv:2102.04164 [cond-mat.mtrl-sci]* **2021**.
